# A real-world nomogram integrating predictive markers for disease-free survival in postmenopausal triple-negative breast cancer receiving anthracycline/taxane-based chemotherapy

**DOI:** 10.3389/fonc.2026.1904801

**Published:** 2026-10-12

**Authors:** Yan Lin, Shao-hong Yu, Chai-Ming Zeng, Shuang-long Cai, Lei Han

**Affiliations:** 1 Department of Geriatric Medicine, Fuzhou University Affiliated Provincial Hospital, Fuzhou, Fujian, China; 2 Fujian Provincial Center for Geriatrics, Fujian Provincial Hospital, Fuzhou, Fujian, China; 3 Peking Union Medical College Hospital, Peking Union Medical College and Chinese Academy of Medical Sciences, Beijing, China; 4 Department of Thyroid and Breast Surgery, Comprehensive Breast Health Center, The Lishui Hospital of Wenzhou Medical University, The First Affiliated Hospital of Lishui University, Lishui People’s Hospital, Lishui, Zhejiang, China; 5 Department of Breast Surgery, Fuzhou University Affiliated Provincial Hospital, Fujian Provincial Hospital, Fuzhou, Fujian, China

**Keywords:** nomogram, postmenopausal, predictive markers, real-world study, triple-negative breast cancer

## Abstract

**Background:**

Triple-negative breast cancer (TNBC) is an aggressive subtype with poor prognosis, especially in postmenopausal women who are routinely treated with anthracycline/taxane-based chemotherapy. However, wide inter-individual variation in disease-free survival (DFS) underscores an urgent need for reliable predictive markers and practical prognostic tools. We aimed to develop and validate a real-world nomogram that integrates clinically accessible predictive markers for individualised DFS prediction in this specific population.

**Methods:**

In this two-centre real-world study, we enrolled 479 postmenopausal TNBC patients who received initial adjuvant anthracycline/taxane-based chemotherapy (sequential or concurrent). Patients were randomly assigned 7:3 to a training cohort (n=335) and a validation cohort (n=144). We used univariate and multivariate Cox regression with stepwise bidirectional selection to identify independent predictors, which were then incorporated into a nomogram. Model performance was assessed using Harrell’s C-index, time-dependent receiver operating characteristic curves, and calibration plots.

**Results:**

Baseline characteristics did not differ significantly between the two cohorts (all P>0.05). Multivariate Cox analysis identified four independent predictors: postoperative pathological T stage (T2+T3 vs T1: hazard ratio [HR]=2.607, 95% CI 1.347-5.047, P = 0.004); N stage (N2 vs N0: HR = 4.077, 2.049-8.112, P<0.001; N3 vs N0: HR = 4.479, 2.561-7.835, P<0.001); high stromal tumour-infiltrating lymphocyte (sTIL) expression (vs low: HR = 0.025, 0.008-0.081, P<0.001); and Ki67 >20% (vs ≤20%: HR = 2.060, 1.082-3.919, P = 0.028). The nomogram achieved excellent discrimination: training cohort C-index=0.899; validation cohort C-index=0.861. Time-dependent AUCs for 1-, 3-, and 5-year DFS were 0.915, 0.945, and 0.959 in the training cohort, and 0.895, 0.943, and 0.900 in the validation cohort, respectively. Calibration curves showed good agreement between predicted and observed outcomes.

**Conclusion:**

We developed and validated a real-world nomogram integrating four readily available predictive markers-T stage, N stage, sTIL, and Ki67-that accurately predicts 1-, 3-, and 5-year DFS in postmenopausal TNBC patients receiving standard anthracycline/taxane chemotherapy. This practical tool supports personalised risk stratification and clinical decision-making, directly addressing the need for predictive markers in advancing breast cancer chemotherapy.

## 1 Introduction

Breast cancer remains the most commonly diagnosed malignancy and the leading cause of cancer-related death amongst women worldwide, with an estimated 2.433 million new cases and 693,000 deaths in 2024 (1). Amongst all molecular subtypes, triple-negative breast cancer (TNBC)-defined by the absence of oestrogen receptor, progesterone receptor, and human epidermal growth factor receptor 2 (HER2) expression-accounts for 15-20% of all breast cancers but contributes disproportionately to recurrence and disease-specific mortality because of its aggressive behaviour and limited targeted treatment options (2, 3). Notably, most patients with TNBC are diagnosed after menopause, and this subgroup often exhibits distinct metabolic and immunological profiles that may affect both treatment response and prognosis (4).

For early-stage TNBC, anthracycline- and taxane-based chemotherapy remains the cornerstone of systemic therapy, whether given neoadjuvantly or adjuvantly (5). Current clinical practice guidelines from ESMO and NCCN recommend these regimens as standard of care (6, 7). Nevertheless, even after optimal chemotherapy, a substantial proportion of patients relapse within 2–3 years, whereas others remain disease-free for extended periods (8). This heterogeneity highlights an urgent clinical need for robust predictive markers that can accurately stratify patients by individual recurrence risk, thereby guiding postoperative surveillance intensity and adjuvant treatment decisions (9).

Traditional clinicopathological parameters-tumour size (T stage), lymph node status (N stage), and histological grade-offer only limited prognostic accuracy when used alone (10). In recent years, extensive research has established stromal tumour-infiltrating lymphocytes (sTIL) as a powerful prognostic and predictive biomarker in TNBC. Large prospective-retrospective analyses of randomised trials consistently show that higher sTIL levels correlate with improved pathological complete response rates and significantly prolonged survival (11–13). Biologically, sTIL reflects pre-existing adaptive antitumour immunity, which enhances chemosensitivity and eradicates micrometastatic disease (14). Similarly, the proliferation marker Ki67 remains one of the most widely available and cost-effective predictors of recurrence risk, although the optimal cutoff value continues to be debated (15, 16).

Beyond traditional factors, emerging evidence suggests that components of the tumour immune microenvironment, cytokeratin expression patterns (e.g., CK5/6), and growth factor receptors such as EGFR may also contribute to TNBC prognosis (17, 18). Furthermore, advances in understanding chemotherapy resistance mechanisms-including alterations in drug targets, apoptotic dysregulation, ABC transporter overexpression, and epithelial-mesenchymal transition-have opened new avenues for biomarker discovery (19, 20). Nevertheless, few existing prognostic models have been specifically developed for postmenopausal TNBC patients receiving uniform, standardised anthracycline/taxane-based regimens with validation using real-world multicentre data. Therefore, using real-world data from two large Chinese tertiary hospitals, we aimed to: (1) characterise DFS patterns in postmenopausal TNBC patients treated with initial anthracycline/taxane chemotherapy; (2) identify independent predictive markers using Cox regression with stepwise selection; (3) construct and validate a practical nomogram to predict 1-, 3-, and 5-year DFS; and (4) visualise survival differences using Kaplan-Meier curves.

## 2 Methods

### 2.1 Study design and participants

This retrospective, two-centre cohort study included postmenopausal women with histologically confirmed TNBC who received initial adjuvant anthracycline/taxane-based chemotherapy at Fuzhou University Affiliated Provincial Hospital (Fujian, China) and Peking Union Medical College Hospital (Beijing, China) between Jan 1, 2016 and Dec 31, 2021(**Figure 1**).

**Figure 1:**
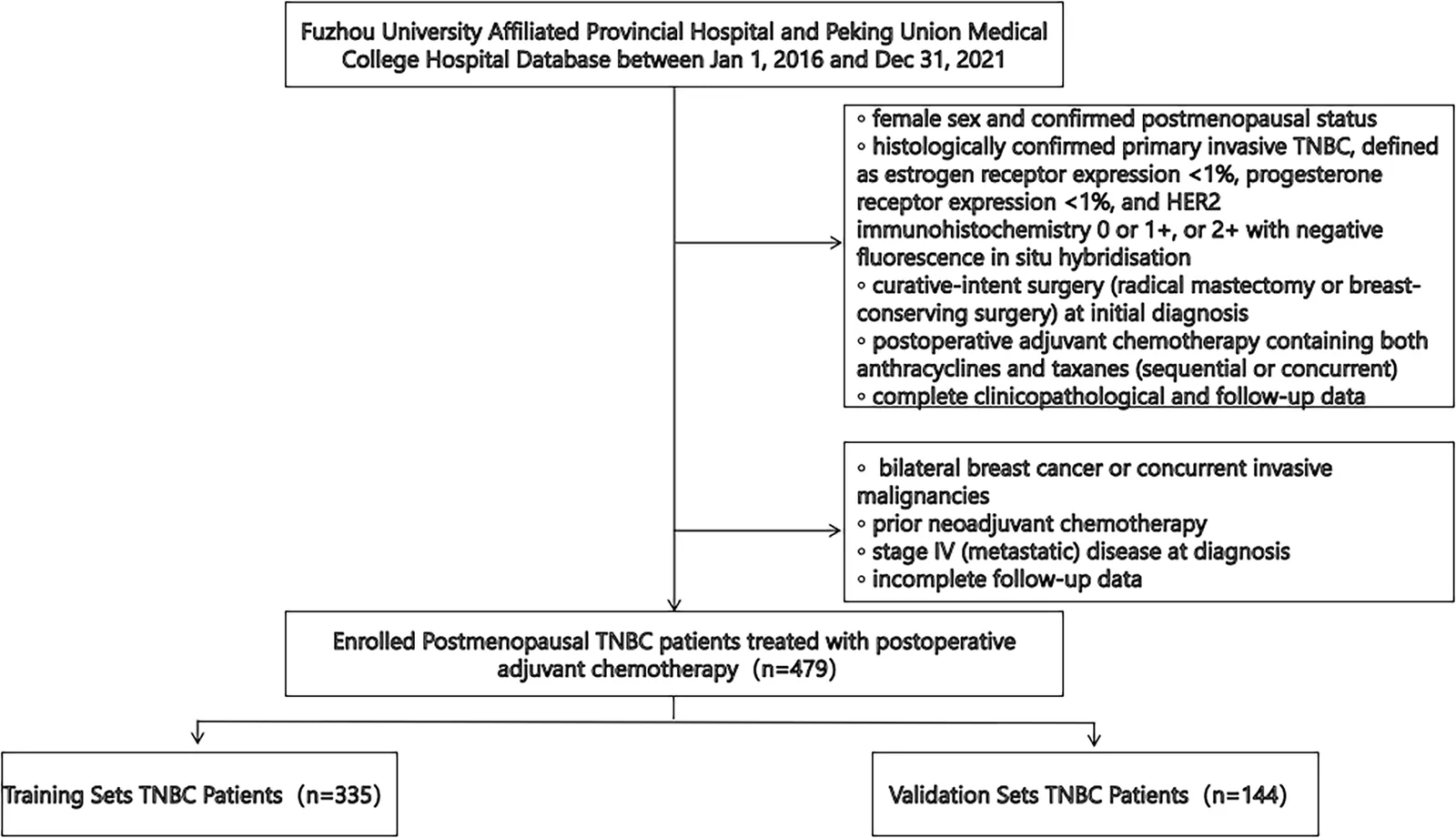
Patient selection flowchart for this study.

Inclusion criteria were: (1) female sex and confirmed postmenopausal status; (2) histologically confirmed primary invasive TNBC, defined as oestrogen receptor expression <1%, progesterone receptor expression <1%, and HER2 immunohistochemistry 0 or 1+, or 2+ with negative fluorescence *in situ* hybridisation; (3) curative-intent surgery (radical mastectomy or breast-conserving surgery) at initial diagnosis; (4) postoperative adjuvant chemotherapy containing both anthracyclines and taxanes (sequential or concurrent); and (5) complete clinicopathological and follow-up data.

Exclusion criteria were: (1) bilateral breast cancer or concurrent invasive malignancies; (2) prior neoadjuvant chemotherapy; (3) stage IV (metastatic) disease at diagnosis; and (4) incomplete follow-up data.

### 2.2 Variable definition and data collection

The following variables were abstracted from the electronic medical records: age at diagnosis; family history; surgical approach; postoperative pathological T stage; postoperative pathological N stage; tumour grade; lymph-vascular invasion; sTIL expression level; HER2 expression level; Ki-67 index; CK5/6 expression; EGFR expression; and receipt of radiotherapy.

HER2 status assessment. HER2 protein expression was assessed by immunohistochemistry (IHC). Equivocal cases (IHC 2+) underwent reflex fluorescence *in situ* hybridisation (FISH) in accordance with American Society of Clinical Oncology/College of American Pathologists (ASCO/CAP) guidelines (21). To minimise inter-institutional variability, a recognised challenge in HER2-low scoring, all IHC and FISH slides were centrally reviewed by two experienced breast pathologists who were masked to patient outcomes. HER2-low status was defined as IHC 1+ or IHC 2+/FISH-negative. For the primary and sensitivity analyses, patients were classified into three categories: HER2 IHC 0, HER2 IHC 1+, and HER2 IHC 2+/FISH-negative.

sTIL assessment. sTILs were evaluated by two experienced pathologists blinded to clinical outcomes, according to international guidelines. sTIL density was categorised into three groups: low (≤10%), intermediate (10–40%), and high (>40%).

### 2.3 Follow-up strategy

Follow-up began on the date of initial breast cancer diagnosis and ended on June 1, 2025. Follow-up was conducted through outpatient visits and telephone interviews. During the first 2 years after surgery, patients were reviewed every 3 months; between years 2 and 5, every 6 months; and beyond 5 years, annually. Routine assessments comprised breast ultrasonography, liver ultrasonography, chest computed tomography (CT), and serum tumour markers (CEA, CA125, and CA153). For patients with suspected bone or brain metastases, whole-body bone scintigraphy and cranial magnetic resonance imaging (MRI) were added, and biopsy was performed when necessary to confirm local or distant recurrence. Data collected included local or regional recurrence, distant metastasis, time to first recurrence or metastasis, site of first recurrence or metastasis, and vital status at the last follow-up.

Disease-free survival (DFS) was defined as the time from the date of surgery to the first occurrence of any of the following events: local recurrence, regional recurrence, distant metastasis, contralateral breast cancer, or death from any cause.

### 2.4 Procedures and data collection

Using a random seed of 208956, we randomly split the patient cohort into a training set (70%, n=335) and a validation set (30%, n=144). We compared categorical variables between cohorts using the χ² test or Fisher’s exact test, and continuous variables using the Mann-Whitney U test.

In the training cohort, we performed univariate Cox proportional hazards regression for each candidate variable. Variables with P<0.05 were entered into a multivariate Cox regression model with stepwise bidirectional selection (based on Akaike information criterion) to identify independent predictive markers of DFS. Hazard ratios (HR) and 95% confidence intervals (CI) were calculated.

For each independent predictor (P<0.05 in multivariate analysis), we plotted Kaplan-Meier survival curves for all patients and compared survival differences using the log-rank test.

We constructed a nomogram based on the independent predictors using the “rms” package in R, assigning points to each predictor proportional to its regression coefficient. Total points were summed to predict 1-, 3-, and 5-year DFS probabilities.

Model discrimination was assessed using Harrell’s C-index and time-dependent area under the curve (AUC) from receiver operating characteristic (ROC) analysis for 1-, 3-, and 5-year DFS. Calibration was evaluated by plotting predicted DFS probabilities against observed Kaplan-Meier estimates at the same time points, with 1000 bootstrap resamples to reduce overfitting. All statistical tests were two-sided, with P<0.05 considered statistically significant. Analyses were performed using R software version 4.2.1 (R Foundation for Statistical Computing, Vienna, Austria).

## 3 Results

### 3.1 Baseline characteristics and cohort comparability

A total of 479 postmenopausal TNBC patients were included in this study. Using a random seed of 208956, patients were randomly assigned at a 7:3 ratio, resulting in 335 patients in the training cohort and 144 patients in the validation cohort. The baseline clinicopathological characteristics of both cohorts are summarised in **Table 1**.

**Table 1:** The baseline clinicopathological characteristics of TNBC patients in both the training and validation sets.

Variables	Total (n=479)	Training Sets (n=335)	Validation sets (n=144)	P value
Age at diagnosis				0.805
<50 years	28 (5.85)	19 (5.67)	9 (6.25)	
≥50 years	451 (94.15)	316 (94.33)	135 (93.75)	
Family history				0.692
No	433 (90.40)	304 (90.75)	129 (89.58)	
Yes	46 (9.60)	31 (9.25)	15 (10.42)	
Surgical approach				0.156
Radical surgery	436 (91.02)	309 (92.24)	127 (88.19)	
Breast-conserving surgery	43 (8.98)	26 (7.76)	17 (11.81)	
Postoperative pathological T staging				0.105
T1	193 (40.29)	127 (37.91)	66 (45.83)	
T2+T3	286 (59.71)	208 (62.09)	78 (54.17)	
Postoperative pathological N staging				0.147
N0	299 (62.42)	203 (60.60)	96 (66.67)	
N1	93 (19.42)	73 (21.79)	20 (13.89)	
N2	42 (8.77)	26 (7.76)	16 (11.11)	
N3	45 (9.39)	33 (9.85)	12 (8.33)	
Tumour grade				0.758
G1+G2	208 (43.42)	147 (43.88)	61 (42.36)	
G3	271 (56.58)	188 (56.12)	83 (57.64)	
Lymph-vascular invasion				0.396
No	350 (73.07)	241 (71.94)	109 (75.69)	
Yes	129 (26.93)	94 (28.06)	35 (24.31)	
sTIL expression levels				0.100
Low	129 (26.93)	92 (27.46)	37 (25.69)	
Intermediate	58 (12.11)	47 (14.03)	11 (7.64)	
High	292 (60.96)	196 (58.51)	96 (66.67)	
Her2 expression levels				0.108
IHC 0	119 (24.84)	86 (25.67)	33 (22.92)	
IHC 1+	259 (54.07)	187 (55.82)	72 (50.00)	
IHC 2+/Fish-	101 (21.09)	62 (18.51)	39 (27.08)	
Ki67				0.393
Ki67 ≤ 20	153 (31.94)	111 (33.13)	42 (29.17)	
Ki67>20	326 (68.06)	224 (66.87)	102 (70.83)	
CK5/6				0.257
Negative	101 (21.09)	66 (19.70)	35 (24.31)	
Positive	378 (78.91)	269 (80.30)	109 (75.69)	
EGFR				0.229
Negative	66 (13.78)	42 (12.54)	24 (16.67)	
Positive	413 (86.22)	293 (87.46)	120 (83.33)	
Radiation therapy status				0.407
No	323 (67.43)	222 (66.27)	101 (70.14)	
Yes	156 (32.57)	113 (33.73)	43 (29.86)	

Family history, HBOC-related cancer history.

sTIL, stromal tumour-infiltrating lymphocytes; IHC, immunohistochemistry. CK5/6, cytokeratin 5/6; HER2, human epidermal growth factor receptor 2; FISH, fluorescence *in situ* hybridization; EGFR, epidermal growth factor receptor.

Importantly, there were no statistically significant differences between the training and validation cohorts across all examined variables: age at diagnosis (P = 0.805), family history (P = 0.692), surgical approach (P = 0.156), postoperative pathological T staging (P = 0.105), postoperative pathological N staging (P = 0.147), tumour grade (P = 0.758), lymph-vascular invasion (P = 0.396), sTIL expression levels (P = 0.100), HER2 immunohistochemistry expression (P = 0.108), Ki67 (P = 0.393), CK5/6 (P = 0.257), EGFR (P = 0.229), and radiation therapy status (P = 0.407). These results confirm that the two cohorts were well-balanced with respect to all measured clinical and pathological characteristics, thereby justifying their use as training and validation cohorts for predictive model development.

The median follow-up for the entire cohort was 82.0 months. In the training cohort (n=335), 87 DFS events (recurrence or death) and 83 deaths occurred during follow-up. In the validation cohort (n=144), 34 DFS events and 34 deaths occurred during follow-up.

### 3.2 Univariate Cox regression analysis

Univariate analysis in the training cohort (**Table 2**) identified the following factors significantly associated with DFS (P<0.05): family history (HR = 2.063, 95% CI 1.144–3.722, P = 0.016); postoperative pathological T2+T3 stage (HR = 4.405, 95% CI 2.393-8.110, P<0.001); postoperative pathological N2 stage (HR = 5.023, 95% CI 2.608-9.672, P<0.001); postoperative pathological N3 stage (HR = 17.255, 95% CI 10.206-29.174, P<0.001); lymph-vascular invasion (HR = 4.589, 95% CI 2.991-7.042, P<0.001); intermediate sTIL (HR = 0.486, 95% CI 0.296-0.799, P = 0.004); high sTIL (HR = 0.013, 95% CI 0.004-0.042, P<0.001); Ki67 >20% (HR = 3.542, 95% CI 1.924-6.520, P<0.001); and radiation therapy (HR = 1.712, 95% CI 1.118-2.620, P = 0.013).

**Table 2:** Univariate Cox regression analysis of disease-free survival for TNBC patients.

Variable	Estimate	Se	z	Wald	p	HR (95% CI)
Age at diagnosis						
<50 years	ref					
≥50 years	1.754	1.006	1.743	3.040	0.081	5.776 (0.804, 41.480)
Family history						
No	ref					
Yes	0.724	0.301	2.405	5.786	0.016	2.063 (1.144, 3.722)
Surgical approach						
Radical surgery	ref					
Breast-conserving surgery	-0.921	0.588	-1.567	2.456	0.117	0.398 (0.126, 1.260)
Postoperative pathological T staging						
T1	ref					
T2+T3	1.483	0.311	4.762	22.674	<0.001	4.405 (2.393, 8.110)
Postoperative pathological N staging						
N0	ref					
N1	0.243	0.334	0.727	0.528	0.467	1.275 (0.663, 2.452)
N2	1.614	0.334	4.828	23.307	<0.001	5.023 (2.608, 9.672)
N3	2.848	0.268	10.629	112.983	<0.001	17.255 (10.206, 29.174)
Tumour grade						
G1+G2	ref					
G3	-0.259	0.216	-1.201	1.444	0.230	0.772 (0.506, 1.178)
Lymph-vascular invasion						
No	ref					
Yes	1.524	0.218	6.974	48.643	<0.001	4.589 (2.991, 7.042)
sTIL expression levels						
Low	ref					
Intermediate	-0.722	0.253	-2.848	8.109	0.004	0.486 (0.296, 0.799)
High	-4.337	0.593	-7.316	53.520	<0.001	0.013 (0.004, 0.042)
Her2 expression levels						
IHC 0	ref					
IHC 1+	-0.390	0.238	-1.639	2.687	0.101	0.677 (0.424, 1.079)
IHC 2+/Fish-	-0.628	0.343	-1.830	3.349	0.067	0.534 (0.272, 1.046)
Ki67						
Ki67 ≤ 20	ref					
Ki67>20	1.265	0.311	4.063	16.505	<0.001	3.542 (1.924, 6.520)
CK5/6						
Negative	ref					
Positive	-0.005	0.271	-0.020	0.000	0.984	0.995 (0.585, 1.691)
EGFR						
Negative	ref					
Positive	-0.497	0.277	-1.792	3.210	0.073	0.609 (0.353, 1.048)
Radiation therapy status						
No	ref					
Yes	0.537	0.217	2.475	6.124	0.013	1.712 (1.118, 2.620)

### 3.3 Multivariate Cox regression and independent predictors

After stepwise bidirectional selection, the final multivariate model (**Table 3**) retained four independent predictive markers:

**Table 3:** Multivariate Cox regression analysis of disease-free survival for TNBC patients.

Variable	Estimate	Se	z	Wald	p	HR (95% CI)
Postoperative pathological T staging						
T1	ref					
T2+T3	0.958	0.337	2.843	8.081	0.004	2.607 (1.347, 5.047)
Postoperative pathological N staging						
N0	ref					
N1	-0.075	0.363	-0.207	0.043	0.836	0.928 (0.455, 1.890)
N2	1.405	0.351	4.004	16.032	<0.001	4.077 (2.049, 8.112)
N3	1.500	0.285	5.257	27.637	<0.001	4.479 (2.561, 7.835)
sTIL expression levels						
Low	ref					
Intermediate	-0.058	0.306	-0.188	0.035	0.851	0.944 (0.518, 1.720)
High	-3.695	0.605	-6.110	37.337	<0.001	0.025 (0.008, 0.081)
Ki67						
Ki67 ≤ 20	ref					
Ki67>20	0.723	0.328	2.201	4.845	0.028	2.060 (1.082, 3.919)

postoperative pathological T stage (T2+T3 vs T1): HR = 2.607, 95% CI 1.347-5.047, P = 0.004.

postoperative pathological N stage (N2 vs N0): HR = 4.077, 2·049-8.112, P<0.001; (N3 vs N0): HR = 4.479, 2.561-7.835, P<0.001.

High sTIL (vs low): HR = 0.025, 0.008-0.081, P<0.001.

Ki67 >20% (vs ≤20%): HR = 2.060, 1.082-3.919, P = 0.028.

### 3.4 Kaplan-Meier survival analysis

Kaplan-Meier curves confirmed significant DFS differences for all four predictors (log-rank P<0.001 for each; **Figures 2**–**5**). Patients with T2+T3 tumours, higher N stage, low sTIL, or Ki67 >20% had significantly worse DFS.

**Figure 2:**
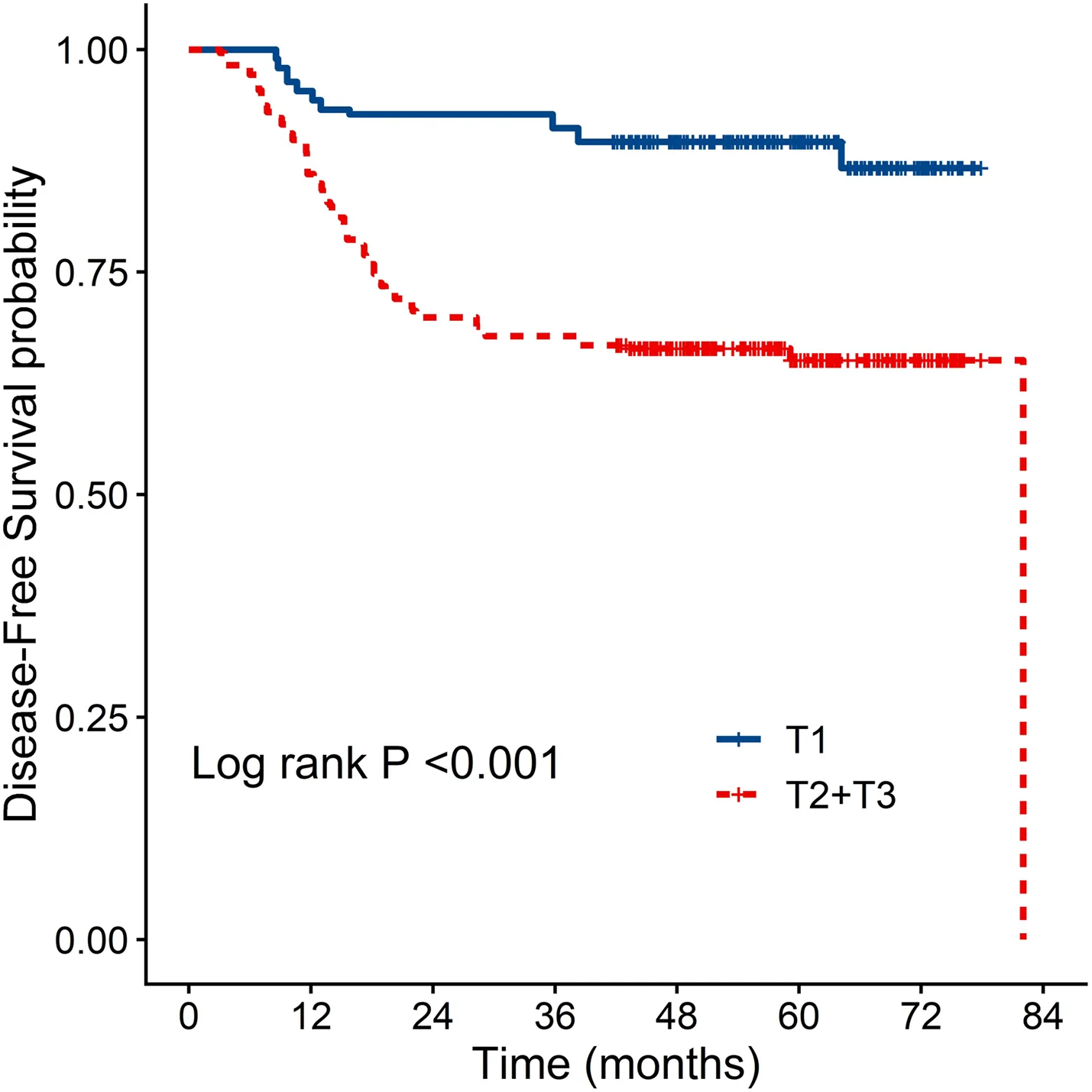
Kaplan-Meier curves for disease-free survival stratified by T staging in all 479 postmenopausal TNBC patients.

**Figure 3:**
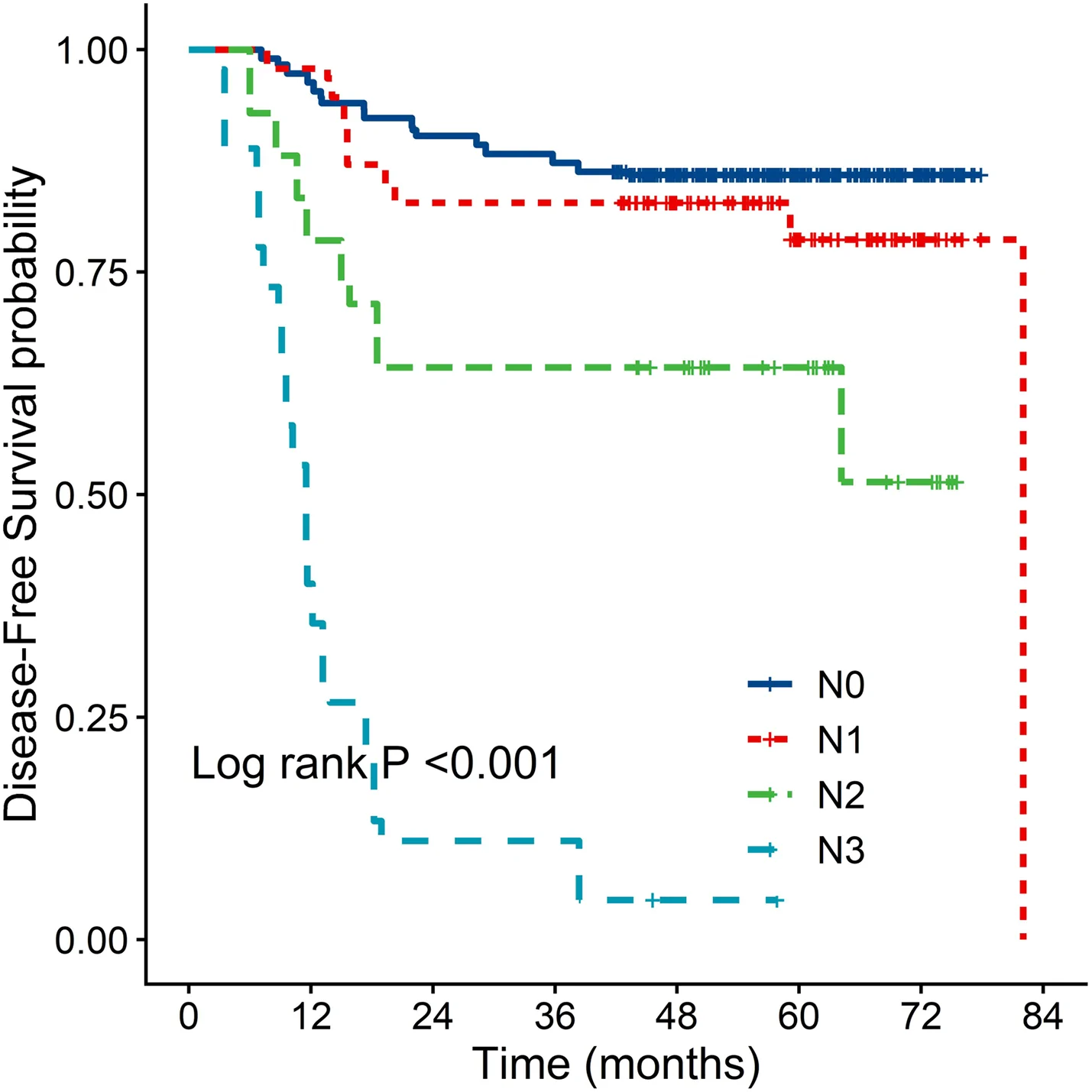
Kaplan-Meier curves for disease-free survival stratified by N staging in all 479 postmenopausal TNBC patients.

**Figure 4:**
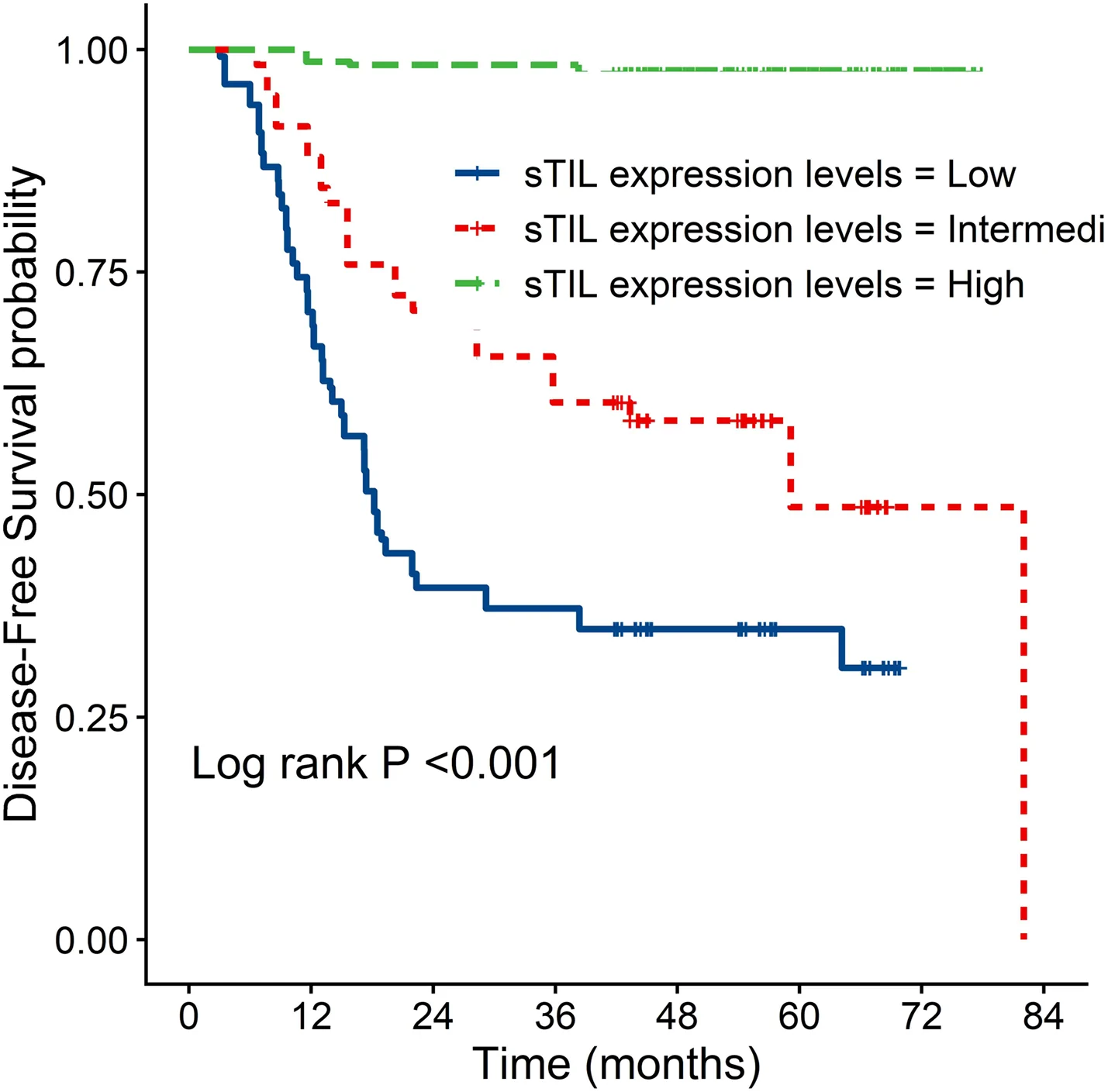
Kaplan-Meier curves for disease-free survival stratified by sTIL expression levels in all 479 postmenopausal TNBC patients.

**Figure 5:**
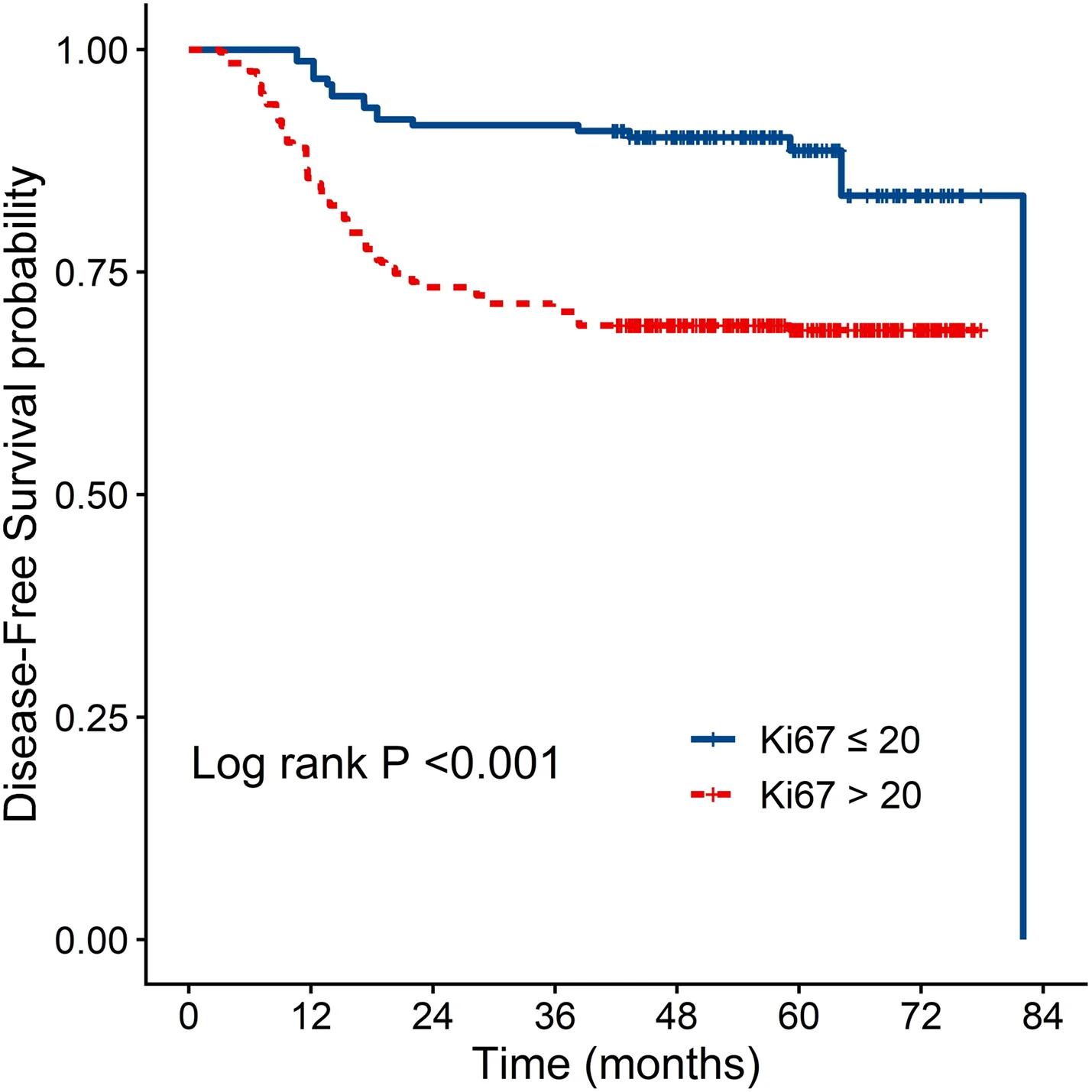
Kaplan-Meier curves for disease-free survival stratified by Ki67 in all 479 postmenopausal TNBC patients.

### 3.5 Nomogram construction

We constructed a nomogram incorporating postoperative pathological T stage, postoperative pathological N stage, sTIL, and Ki67 to predict 1-, 3-, and 5-year DFS probabilities for individual patients (**Figure 6**). Each variable contributes points proportional to its regression coefficient; the total points correspond to predicted DFS at each time point.

**Figure 6:**
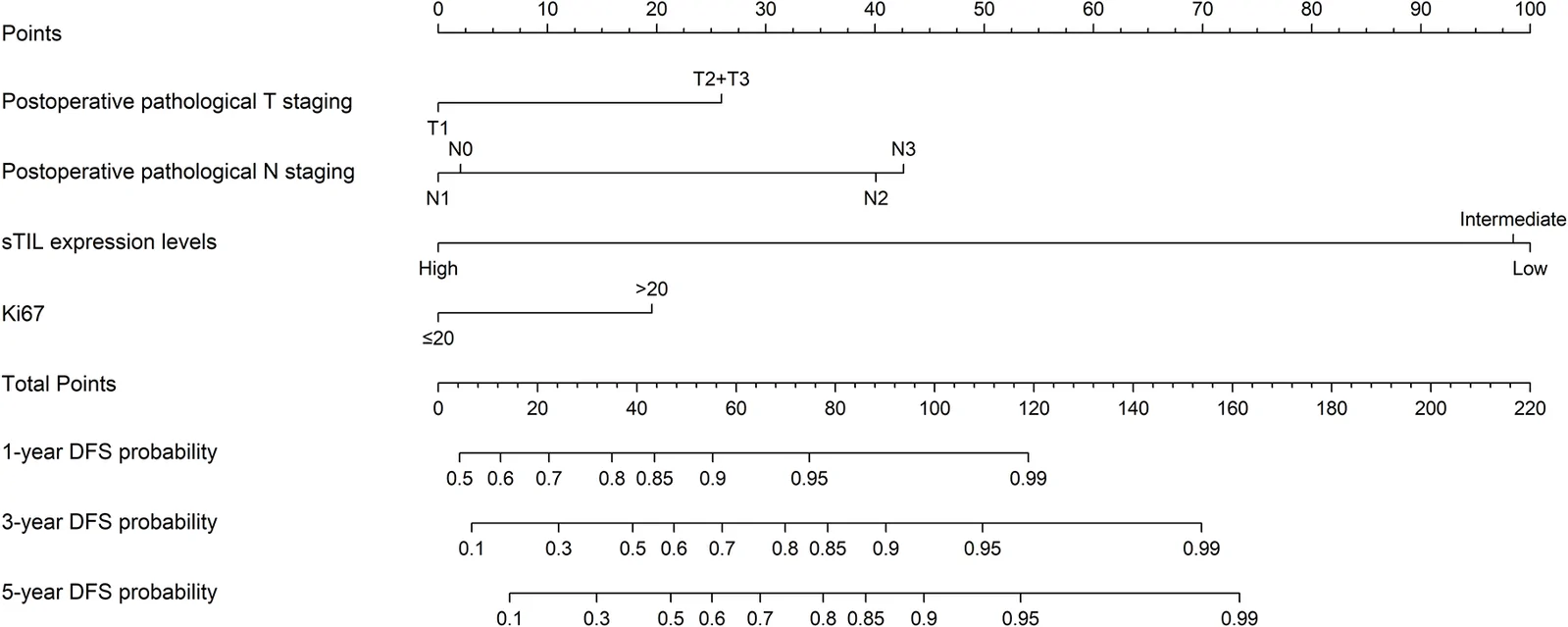
Nomogram integrating four predictive markers for 1-, 3-, and 5-year DFS prediction in postmenopausal TNBC patients.

### 3.6 Model performance and validation

Discrimination: The nomogram achieved excellent discrimination with a C-index of 0.899 in the training cohort and 0.861 in the validation cohort. Time-dependent ROC AUCs for 1-, 3-, and 5-year DFS were 0.915, 0.945, and 0.959 in the training cohort (**Figure 7**) and 0.895, 0.943, and 0.900 in the validation cohort (**Figure 8**).

**Figure 7:**
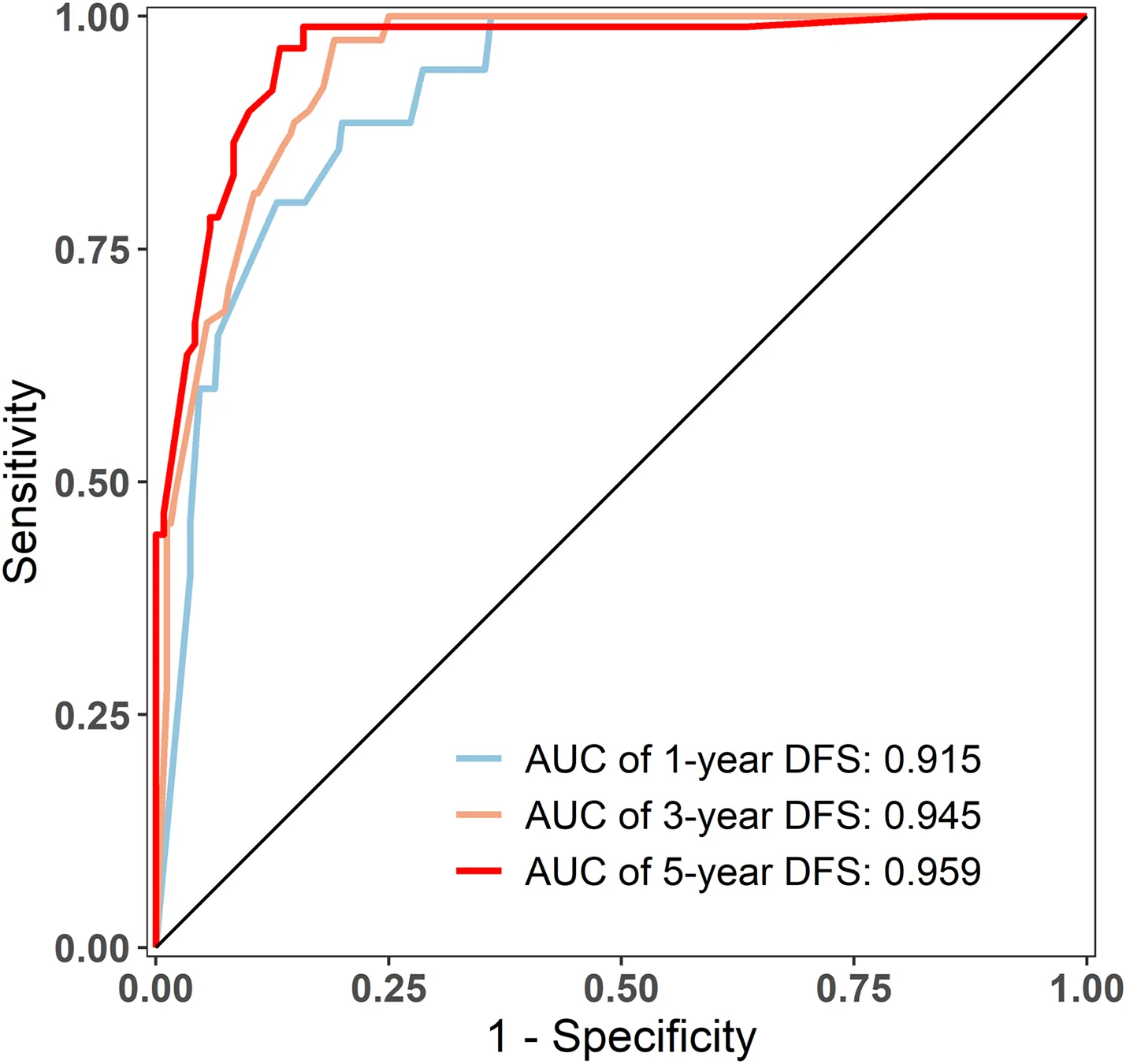
ROC curve of the training set.

**Figure 8:**
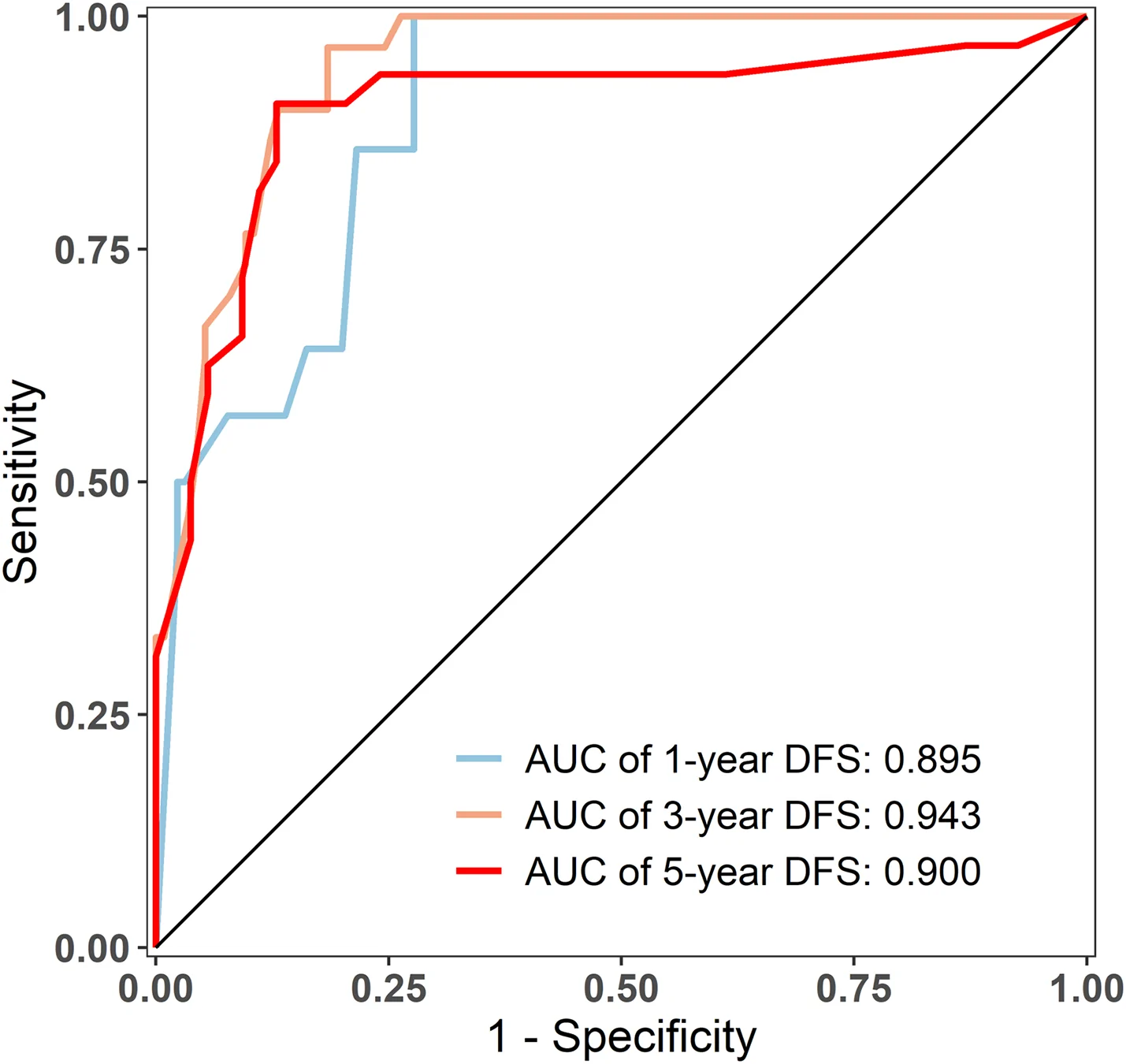
ROC curve of the validation set.

Calibration: Calibration plots showed good agreement between predicted and observed DFS probabilities at 1, 3, and 5 years in both cohorts (**Figures 9**–**14**).

**Figure 9:**
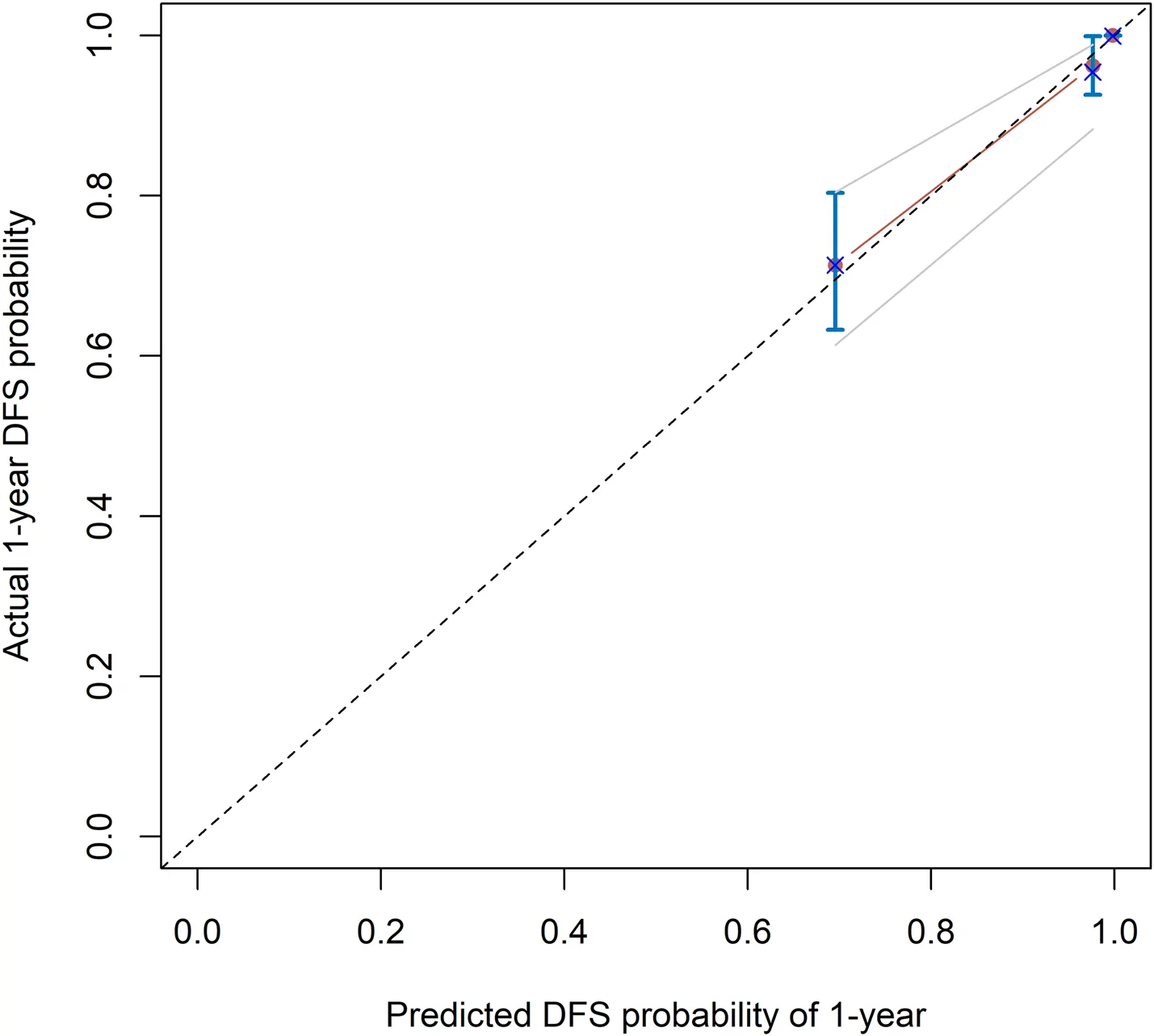
Calibration curve of the training set at 1 year.

**Figure 10:**
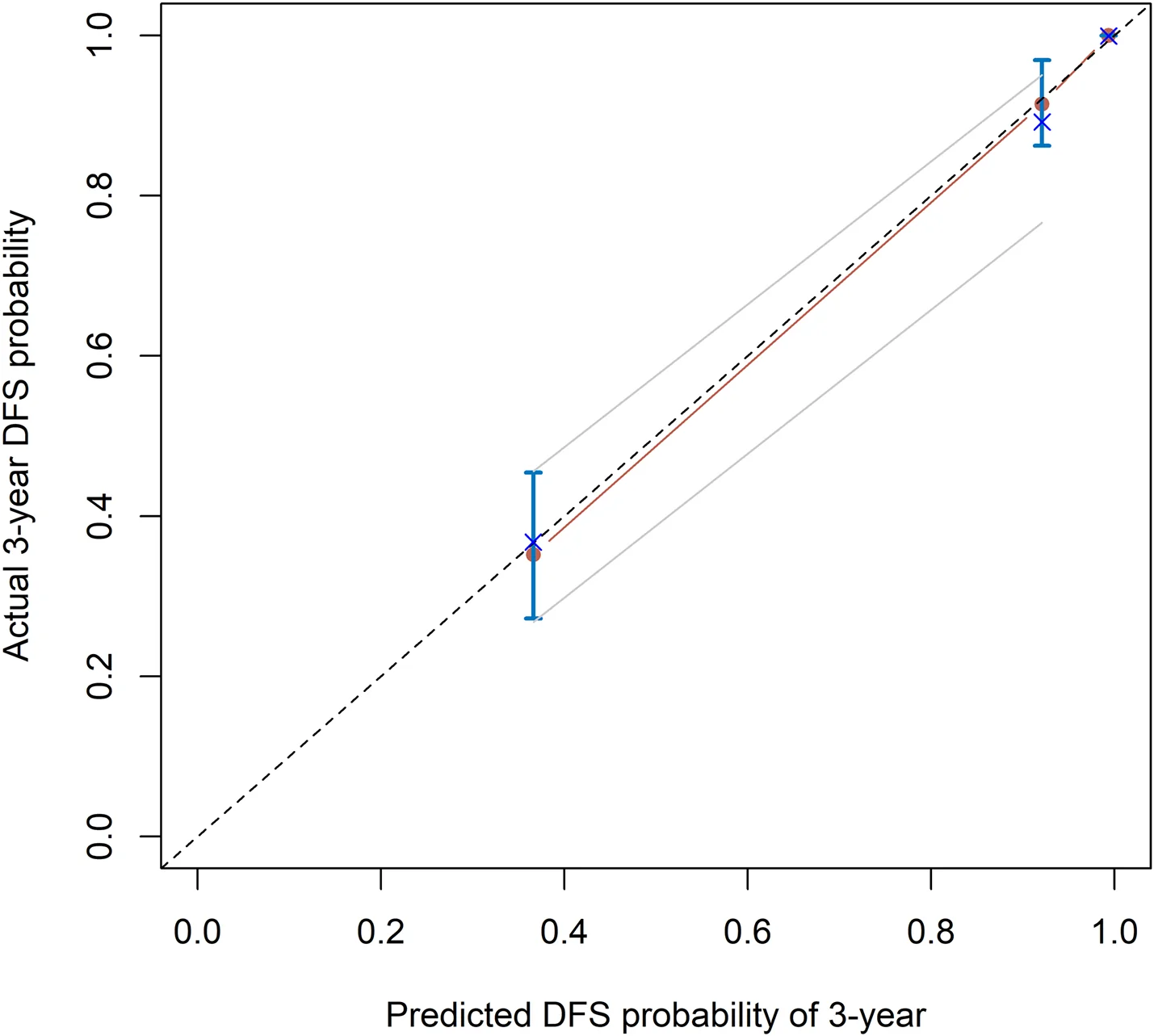
Calibration curve of the training set at 3 year.

**Figure 11:**
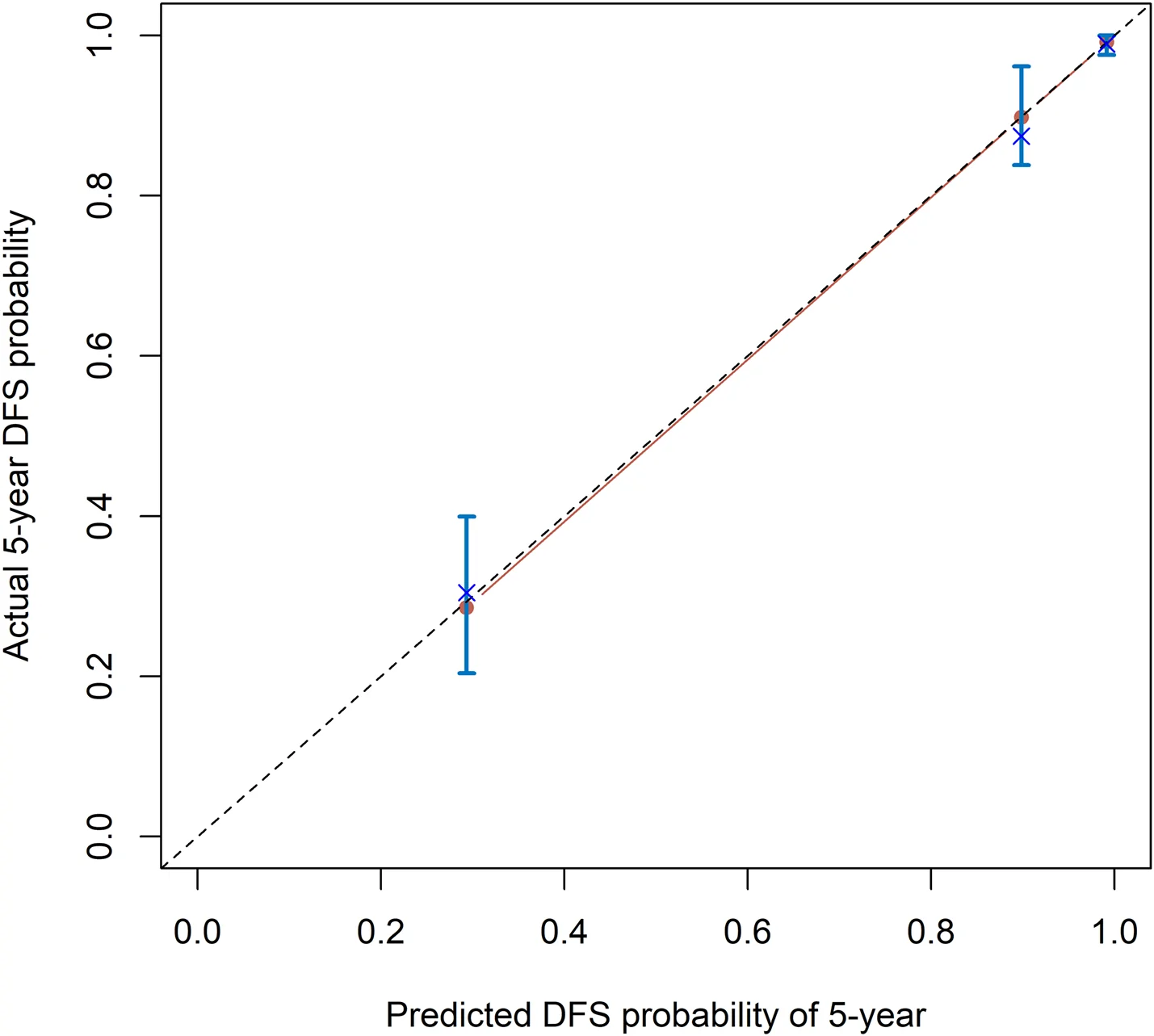
Calibration curve of the training set at 5 year.

**Figure 12:**
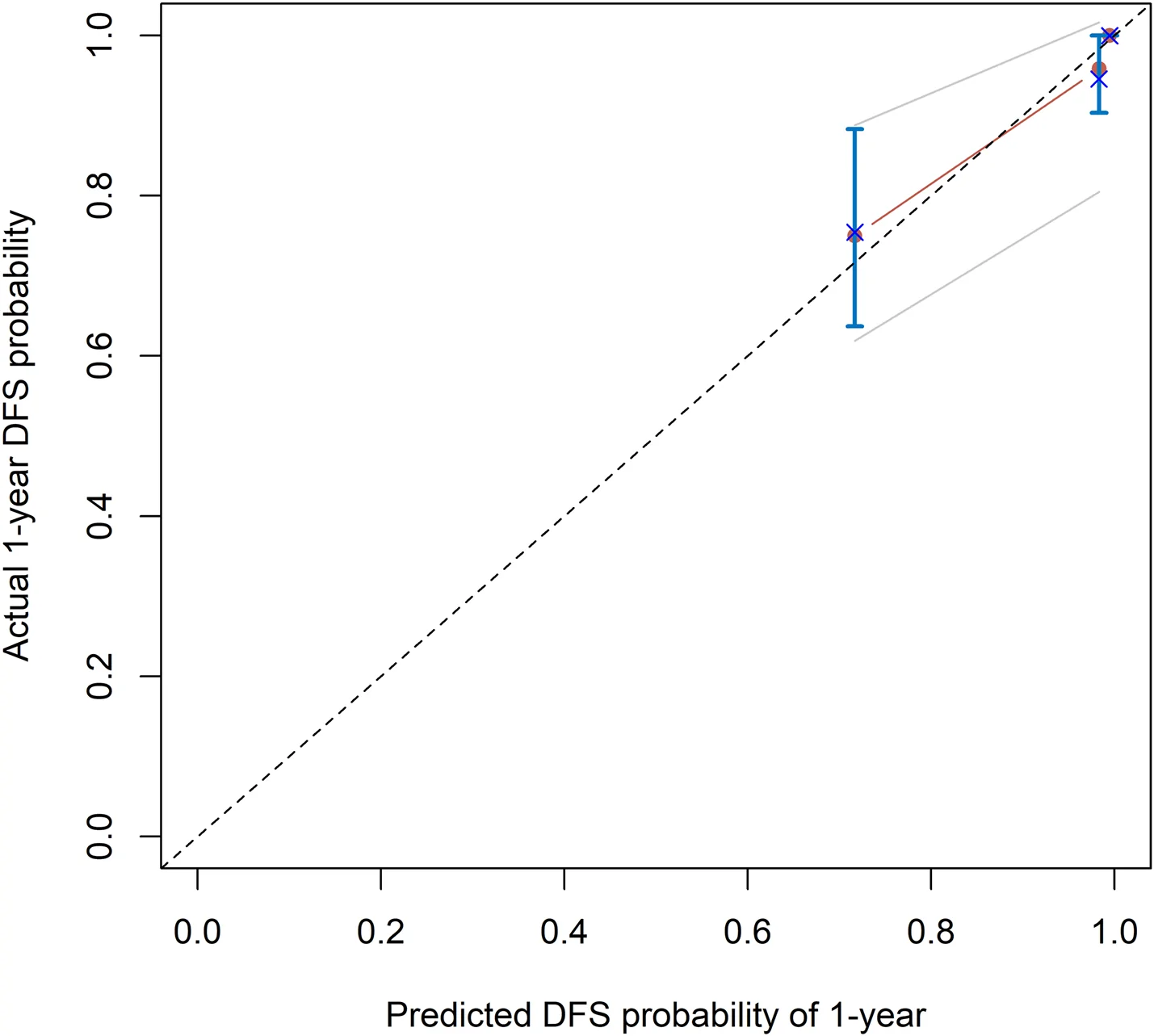
Calibration curve of the validation set at 1 year.

**Figure 13:**
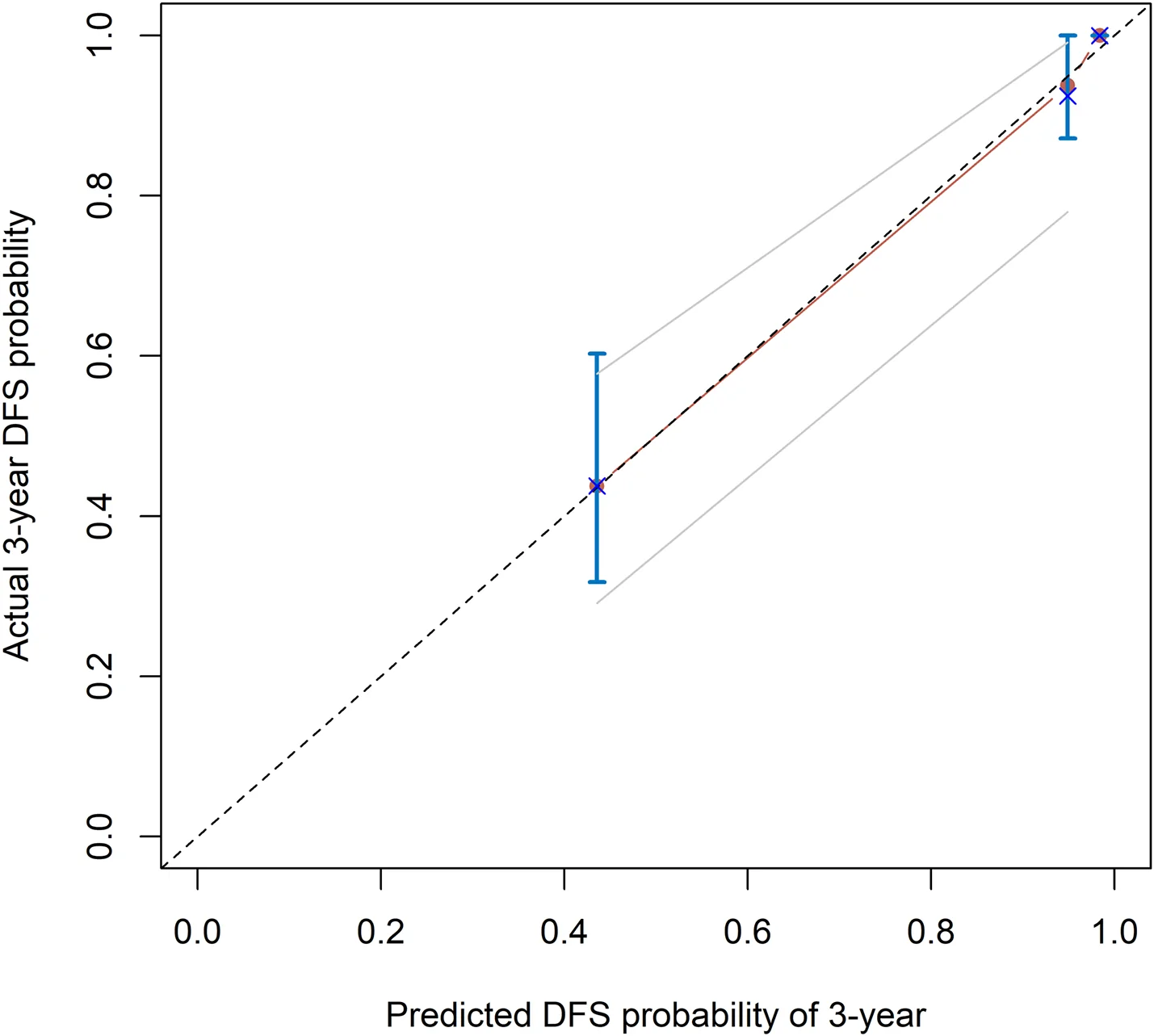
Calibration curve of the validation set at 3 year.

**Figure 14:**
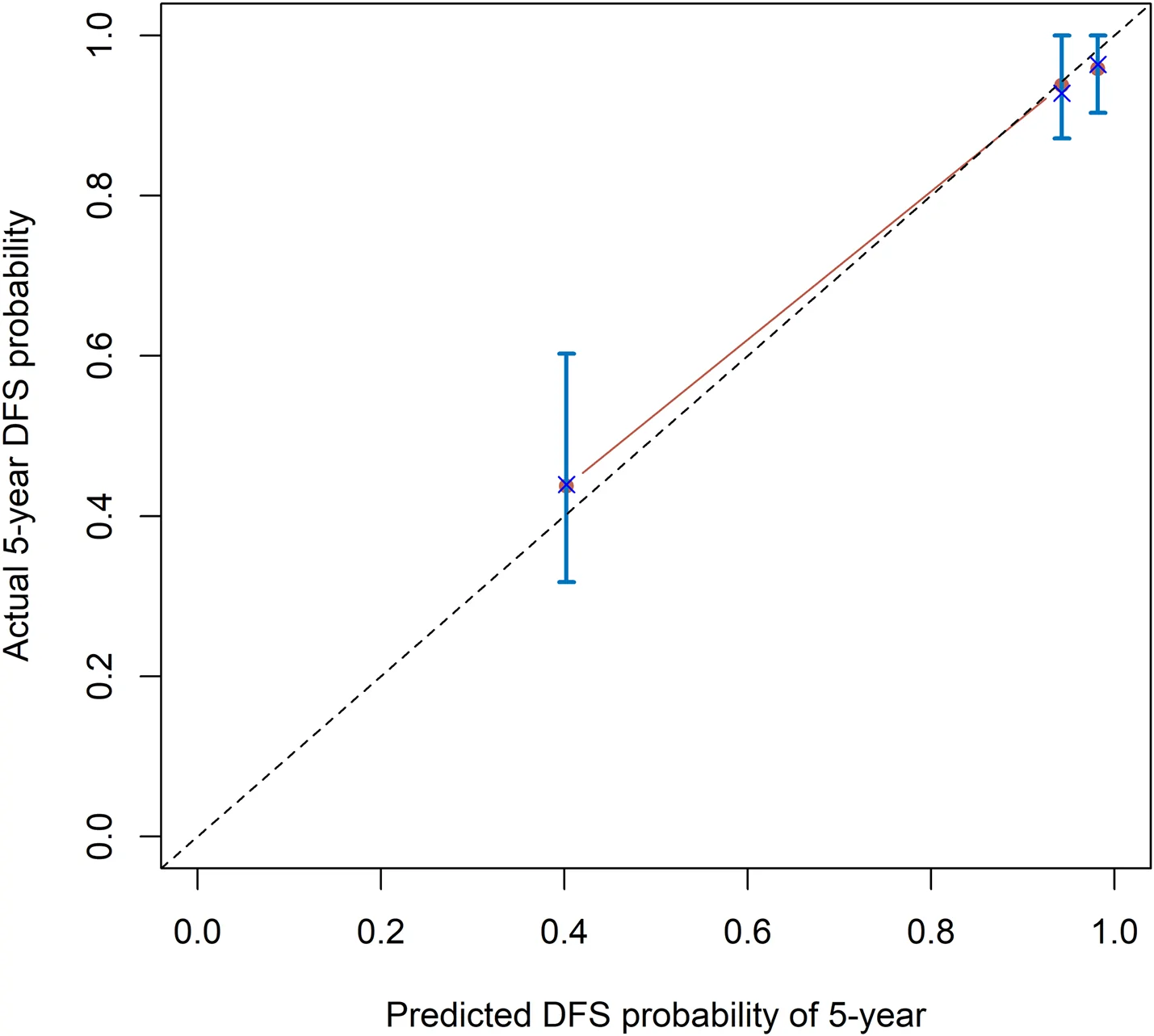
Calibration curve of the validation set at 5 year.

## 4 Discussion

In this multicentre real-world study of 479 postmenopausal TNBC patients treated with uniform, standardised initial anthracycline/taxane-based chemotherapy, we developed and validated a simple yet robust prognostic nomogram based on four readily available markers: postoperative pathological T stage, postoperative pathological N stage, sTIL expression, and Ki67 index. Our model demonstrated excellent discrimination (AUCs consistently >0.89 across all time points in both cohorts) and good calibration, offering clinicians a practical tool for individualised post-chemotherapy risk assessment.

Several prognostic models have been developed for TNBC, although most were not specifically designed for postmenopausal patients receiving uniform anthracycline/taxane-based chemotherapy. Yang YP and colleagues developed nomograms for DFS and OS in Chinese TNBC patients based on stromal TILs, tumour size, node status, and Ki67 index, with a training cohort of 296 patients and external validation in 191 patients; the C-index for DFS was 0.743 in the training cohort and 0.784 in the validation cohort (22). This model shares two predictors with ours (sTIL and Ki67) but did not incorporate T stage or focus on postmenopausal women. A more recent SEER-based study by Gao HY et al. analysed 2,978 TNBC patients and constructed a nomogram incorporating race, AJCC N/M stage, tumour size, surgery type, and pathological response, achieving C-indices of 0.780 and 0.773 in the training and validation cohorts, respectively (23). However, this model relied on pathological response after neoadjuvant chemotherapy and did not include immune or proliferation markers such as sTIL or Ki67. Another SEER-based nomogram by Feng RG et al. specifically targeted elderly women with TNBC (n=5,317) and predicted overall survival (OS) and breast cancer-specific survival (BCSS) using age and clinicopathological factors, with C-indices of 0.797 and 0.825 in the training cohort (24). Although this study addressed an older population, it did not restrict to postmenopausal status, did not incorporate sTIL, and was based on registry data rather than real-world multicentre cohorts with uniform chemotherapy. In contrast, our nomogram integrates four readily available markers-T stage, N stage, sTIL, and Ki67-in a well-defined population of postmenopausal TNBC patients receiving standardised anthracycline/taxane-based chemotherapy, and achieved a C-index of 0.899 in the training cohort and 0.861 in the internal validation cohort. Compared with existing models, our approach offers the advantages of a homogeneous treatment background, inclusion of an immune biomarker (sTIL) with a clear threshold effect, and validation in a real-world two-centre Chinese cohort. We acknowledge, however, that external validation in broader populations and comparison with molecularly informed models remain necessary.

The exceptionally strong protective effect of high sTIL expression (HR = 0.025, P<0.001) aligns with and extends the growing body of evidence positioning sTIL as a critical biomarker in TNBC (11, 12, 25). The pooled analysis by Denkert and colleagues (3,771 patients from six neoadjuvant trials) showed that each 10% increment in sTIL was associated with longer DFS in TNBC (11). Importantly, our analysis revealed that only “high” sTIL-not “intermediate”-retained independent significance, suggesting a threshold effect. This observation is consistent with recent data indicating that dense lymphocytic infiltration is required for meaningful antitumour activity (26). Mechanistically, sTIL reflects pre-existing adaptive immunity, with CD8^+^ cytotoxic T cells mediating chemotherapy-induced tumour cell death and eliminating residual micrometastatic disease (14, 27). The particularly large effect size in our cohort may relate to the postmenopausal setting, where age-related immune changes could influence TIL composition and function (4). These findings support routine sTIL reporting for postmenopausal TNBC patients, as advocated by the International TILs Working Group (28).

Ki67 >20% was independently associated with a two-fold increased recurrence risk (HR = 2.060, P = 0.028). This finding aligns with extensive evidence that high proliferative activity correlates with aggressive tumour behaviour and early relapse in breast cancer (15, 29). A systematic review by Penault-Llorca and colleagues confirmed Ki67 as a robust prognostic marker in TNBC (16), and large cohort studies have further validated that high Ki67 expression predicts worse DFS and overall survival (30, 31). Our results extend these observations to the postmenopausal setting, confirming that Ki67 retains prognostic significance in this specific subgroup receiving uniform anthracycline/taxane chemotherapy.

Despite growing emphasis on molecular and immune biomarkers, our results reaffirm that conventional anatomical staging-particularly N stage-remains a powerful predictor. Patients with N3 disease had a nearly 4.5-fold higher recurrence risk than those with N0 disease, even after multivariable adjustment. This is consistent with a systematic review and meta-analysis by Ambrosini-Spaltro and colleagues, which confirmed lymph node status as an independent prognostic factor for DFS in breast cancer, whereas ER, PR, and HER2 status showed no significant prognostic value (32). More recent SEER-based studies have further validated the prognostic significance of nodal burden across breast cancer subtypes, including TNBC (33). Notably, N1 stage did not retain significance in our multivariate model, likely because the prognostic impact of limited nodal involvement (1–3 positive nodes) is partially attenuated by effective chemotherapy and other competing risk factors (34).

It is noteworthy that lymph-vascular invasion and radiation therapy status, both significant in univariate analysis, were no longer independent predictive markers in the multivariate model. The loss of significance for lymph-vascular invasion can be attributed to its strong collinearity with N stage, as lymph-vascular invasion frequently coexists with axillary nodal metastasis (35).Similarly, In univariate analysis, radiotherapy was associated with an HR of 1.712 (P = 0.013). This apparently adverse association should be interpreted with caution and is most likely explained by confounding by indication: patients with more advanced T or N stage were more likely to receive post-mastectomy radiotherapy (36). Thus, the univariate estimate reflects the higher baseline recurrence risk of patients selected for radiotherapy rather than a harmful effect of radiotherapy itself. After multivariable adjustment, radiotherapy was no longer an independent predictor of DFS.

## 5 Study limitations

This study has several limitations. First, the retrospective design introduces inherent selection bias despite multivariate adjustment; prospective validation is required. Second, sTIL assessment was performed by local pathologists without centralised review. Although we followed International TILs Working Group guidelines, inter-observer variability remains a concern. Future studies should incorporate digital pathology and artificial intelligence-based TIL quantification. Third, our study population was restricted to postmenopausal women of Chinese ethnicity treated at two large urban academic centres. Generalisability to premenopausal patients, other ethnic groups, or community hospital settings requires further investigation. Fourth, the validation cohort was derived by random split from the same two-centre dataset and therefore represents internal rather than true external validation. The validation cohort size is also relatively modest. Larger external validation studies, ideally involving multiple centres, countries, and healthcare settings, are needed to confirm the generalisability of our nomogram before broad clinical implementation. Fifth, TNBC is biologically heterogeneous, and several molecular classification systems, including the Lehmann subtypes (37) and the Fudan classification (38), have been proposed. Because routine molecular subtyping data were not available for all patients in this real-world cohort, TNBC was treated as a single entity in the primary analysis. It is therefore possible that the prognostic value of sTIL, Ki67, T stage, and N stage differs across molecular subtypes, and that the distribution of subtypes could influence model performance. Future studies should incorporate molecular subtyping, transcriptomic profiles, and immune signatures to refine risk stratification and to determine whether the nomogram performs equally well across TNBC subtypes. Finally, anthracycline/taxane regimens were not fully standardised (some patients received sequential, others concurrent), although both are considered standard with comparable efficacy (6).

## 6 Future perspectives

Several promising avenues exist for improving predictive modelling in TNBC. First, integrating dynamic markers such as postoperative circulating tumour DNA (minimal residual disease) could complement our static nomogram with real-time risk assessment. Second, machine learning approaches incorporating high-dimensional data (e.g., genomic, transcriptomic, or radiomic features) may outperform traditional Cox-based nomograms, although interpretability remains a challenge. Third, the rapid evolution of antibody–drug conjugates (e.g., sacituzumab govitecan,trastuzumab deruxtecan) and immunotherapies will likely modify the prognostic significance of traditional markers, necessitatingmodel updating (39). Fourth, given our finding of a threshold effect for sTIL, future studies should explore whether combining low sTIL with other immune-related markers (e.g., PD-L1, CD8 density, or interferon-γ signature) can better identify patients who may benefit from immune checkpoint inhibition.

## 7 Conclusion

We developed and validated a real-world nomogram that integrates four readily available predictive markers-postoperative pathological T stage, N stage, sTIL expression level, and Ki67 index-to accurately predict 1-, 3-, and 5-year DFS in postmenopausal TNBC patients receiving initial anthracycline/taxane-based chemotherapy. The nomogram showed excellent discrimination and good calibration in both training and validation cohorts. This practical tool offers clinicians a user-friendly means of individualising recurrence risk assessment and may inform post-chemotherapy surveillance intensity and treatment decisions. Further prospective validation and incorporation of emerging molecular markers are warranted.

## Data Availability

The original contributions presented in the study are included in the article/supplementary material. Further inquiries can be directed to the corresponding authors.
